# Persistence of Low Pathogenic Influenza A Virus in Water: A Systematic Review and Quantitative Meta-Analysis

**DOI:** 10.1371/journal.pone.0161929

**Published:** 2016-10-13

**Authors:** Antonia E. Dalziel, Steven Delean, Sarah Heinrich, Phillip Cassey

**Affiliations:** 1 School of Biological Sciences, The University of Adelaide, Adelaide, South Australia; 2 Centre for Conservation Science & Technology, The University of Adelaide, Adelaide, South Australia; SWEDEN

## Abstract

Avian influenza viruses are able to persist in the environment, in-between the transmission of the virus among its natural hosts. Quantifying the environmental factors that affect the persistence of avian influenza virus is important for influencing our ability to predict future outbreaks and target surveillance and control methods. We conducted a systematic review and quantitative meta-analysis of the environmental factors that affect the decay of low pathogenic avian influenza virus (LPAIV) in water. Abiotic factors affecting the persistence of LPAIV have been investigated for nearly 40 years, yet published data was produced by only 26 quantitative studies. These studies have been conducted by a small number of principal authors (n = 17) and have investigated a narrow range of environmental conditions, all of which were based in laboratories with limited reflection of natural conditions. The use of quantitative meta-analytic techniques provided the opportunity to assess persistence across a greater range of conditions than each individual study can achieve, through the estimation of mean effect-sizes and relationships among multiple variables. Temperature was the most influential variable, for both the strength and magnitude of the effect-size. Moderator variables explained a large proportion of the heterogeneity among effect-sizes. Salinity and pH were important factors, although future work is required to broaden the range of abiotic factors examined, as well as including further diurnal variation and greater environmental realism generally. We were unable to extract a quantitative effect-size estimate for approximately half (50.4%) of the reported experimental outcomes and we strongly recommend a minimum set of quantitative reporting to be included in all studies, which will allow robust assimilation and analysis of future findings. In addition we suggest possible means of increasing the applicability of future studies to the natural environment, and evaluating the biological content of natural waterbodies.

## Introduction

An organism’s persistence depends on it being capable of surviving the extremes of the prevailing environmental conditions [1]. Viruses are often capable of naturally persisting in a wide variety of environments, including water [2], and can remain infective for varying lengths of time. Viruses, which are able to persist in the environment, may transmit to new hosts, including new types of host, when the opportunity and circumstances arise [2]. A notable example is the megavirus *Pithovirus sibericum*, which was recently isolated from the Siberian permafrost, dated to being more than 30,000 years old, and still infectious on thawing [3]. The environmental conditions that are conducive to viral persistence and transmission are variable, depending on the type of virus and the protection they possess. Physical protection of a virus is provided by its’ capsid protein coat, present in all virions (viral particles) [4, 5], and non-enveloped viruses (in particular) are more resistant to environmental degradation than enveloped viruses [6].

Influenza viruses (*Orthomyxoviridae*) are enveloped, single stranded, negative sense RNA viruses and are divided into four types: influenza A, which infect both avian and mammalian hosts; influenza B, which circulate in humans, and have been isolated from seals [7]; influenza C, found in humans, pigs and marine mammals [8], and recently influenza D, found in cattle and pigs [9]. Influenza A is the largest group of influenza viruses, with recognised differentiation of individual types through the two glycoproteins located on their surface [10, 11]; haemagglutinin (HA, or H-type) and neuraminidase (NA, or N-type). To date, there are sixteen HAs and nine NAs that have been identified from their natural waterbird hosts (predominantly Anseriformes, although Charadriiformes are also known hosts [12, 13]), with multiple strains arising within each HA and NA combination [8].

Outbreaks of influenza in live bird markets [14], zoonotic infections of humans [15, 16], and pandemic influenza events in the last twenty years have each highlighted the wide range of species susceptible to influenza A viruses [17–19]. Considerable research effort has focused on low pathogenic avian influenza viruses (LPAIV), which naturally occur in wild birds, contrasted with highly pathogenic avian influenzas (HPAIV), which have high mortality rates in poultry [20]. LPAIVs circulate within their wild waterbird hosts, whereas HPAIVs are believed to mostly arise after multiple passages through domesticated poultry [21, 22]. LPAIVs are found in both the respiratory and gastrointestinal system of their natural hosts. Waterbirds spend considerable time on or around water, eating, preening and defaecating, with a single duck producing 7.5-10kg of faeces per year [23]. LPAIVs are most commonly shed into the aquatic environment in large volumes via waterbird faeces [24, 25].

Viruses are transmitted, either directly by host to host contact, or indirectly by air, fomites, or environmental contamination[1, 2]. Transmission of LPAIVs includes an environmental component [26, 27], which enables indirect transfer of virus between hosts [28]. The length of time LPAI virus can remain infective in the environment, the specific conditions of the environment that are conducive to persistence, and the infective dose required for transmission [4], have all been the subject of nearly 40 years investigation. As a notifiable disease to both WHO (World Health Organisation) and FAO (Food and Agriculture Organisation of the United Nations), avian influenza is of global importance [29]. Control of LPAIV, and the prevention of disease outbreaks, requires an accurate understanding of: (i) the spread and transmission of the virus both among waterbirds, and between waterbirds and other potential reservoir species (e.g., shorebirds, poultry, pigs, horses, cats and humans); and (ii) the survival and persistence of the virus within the environment, prior to, and facilitating, novel transmission.

Webster et al. (1978) provided the first published quantitative information on the survival of influenza A virus in water, and showed that the virus persisted for up to 30 days, three times longer than in faeces [30]. Their foundational work has been followed by multiple studies, and thirty years after Webster’s initial study Brown et al. (2009) noted that the majority of previous investigations had concentrated on laboratory-based investigations, using distilled water in most cases. This subsequently highlighted the need for broader testing of the properties that affect the survival/persistence of the virus. Laboratory-based studies have provided information regarding persistence of twelve out of sixteen identified HA types (see below) in varying simulated environmental conditions. In the past decade, environmental water samples have been more regularly used in laboratory based studies, including samples from water bodies with known populations of waterbirds, and known circulating LPAIVs in the hosts [31–35]. Two reviews, which synthesised the available information on LPAIV persistence, were previously published by Irwin [36] and Stallknecht [17]. In the most part they agreed with the observed findings from previous individual studies, although notably Irwin did not find temperature to be an important moderator of virus half-life in water, possibly due to the final sample size (7 studies, 127 data points) included for analysis. Meta-analytic review techniques and statistical packages have evolved greatly and it is now possible to examine quantitative relationships, in the persistence of LPAIV across studies (controlling for replicate observations), and the contributions of these variables in explaining persistence of LPAIV.

How the virus interacts with, and is affected by, the environment is a crucial component to understanding the circulation and transmission, particularly if we wish to improve targeting of surveillance and future disease control. Information regarding the environmental persistence of LPAIV is spread across the primary scientific literature. In this study we conducted the first quantitative meta-analysis of the environmental factors that influence the persistence of LPAIV in water. We surveyed and assimilated all of the available literature, in order to provide a comprehensive analysis of the environmental variables previously investigated, and to draw robust conclusions and inferences regarding the persistence of LPAIV in water. We specifically investigated the survival of the virus in water (c.f. [36]), as the natural hosts are intimately associated with, and shed the virus into, a wide range of natural water bodies [37], and LPAIV has been previously isolated from open water [38]. The persistence of the virus in water can be difficult to measure, and so in most studies has been quantified as infectivity to hosts. This is predicated on the virus occurring at an adequate concentration to infect a host, and therefore having a biological effect.

Our objectives were threefold. First, we have identified which of the most commonly studied environmental variables (i.e., temperature, pH, salinity and water type) have a consistent influence on persistence of LPAIV in water. By summarising quantitative results across a broad range of environmental information, and from all known studies, we were able to conduct our analyses for an increasingly realistic range of values. Second, we have investigated the size (and influence) of the effect that these environmental variables have on persistence of LPAIV in water, expanding on the previous reviews and allowing for a greater understanding of the effect in different water-body types. This allows for a more robust translation (and prediction) of the effects of persistence in novel environments, as well as under the potentially altered conditions of climate change [39]. Finally, we have highlighted obvious knowledge gaps among the previously investigated environmental variables, and discussed future priorities for research, including some of our own recommendations for conducting trials that more closely mimic the natural environment.

## Methods

### Literature search

**Fig 1:**
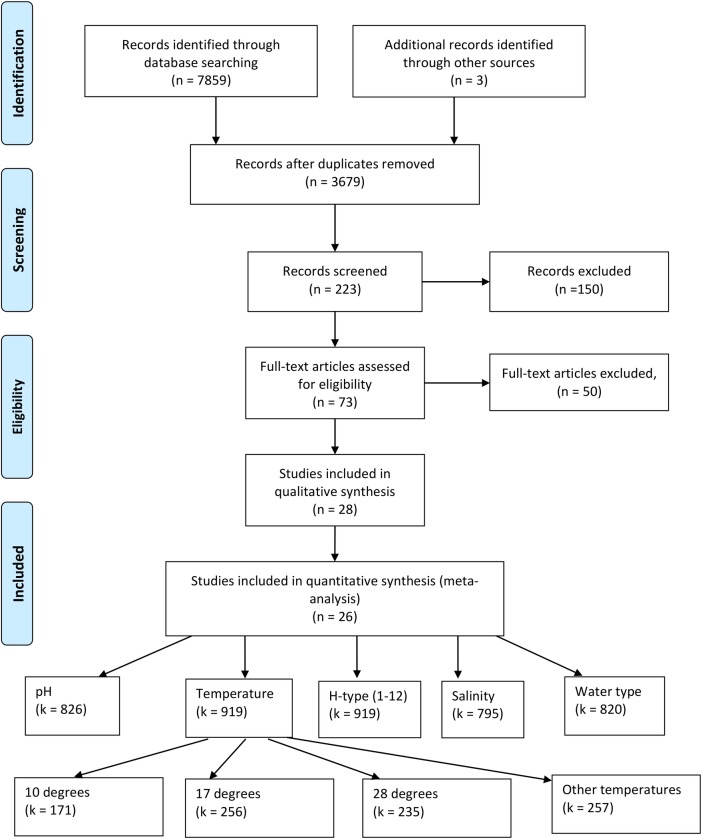
Systematic review PRISMA chart[40]. The chart illustrates the inclusion/exclusion process of reviewing studies and the numbers of papers identified at each stage (n). The forward and backward reference search was conducted at the eligibility stage. The number of samples (k) are provided for the five key variables: temperature, pH, water type, H-type and salinity. An example of the temperatures and number of observations is also given.

We used the systematic review framework, PRISMA [40], to conduct a quantitative meta-analysis of published studies on the environmental factors affecting the persistence of LPAIV in water. We searched four databases (Web of Science, which itself encompasses multiple databases; Aquatic Sciences and Fisheries Abstracts; PubMed; and GoogleScholar), for primary scientific studies of the environmental factors that have been studied in relation to persistence of LPAIV. The search terms were chosen to be as broad as possible, whilst keeping the specific search objectives within reasonable bounds. We used the following search terms: (infl* OR orthomyx*) AND (avian OR bird) AND (surviv* OR persis*). We included studies referred to in two previously published literature surveys [17, 36], if they were not already identified in the search, and completed a wide forwards (citations within a relevant paper) and backwards (citations of a relevant paper) search, including the reference lists of any papers that met our inclusion criteria (see Fig 1 and details below).

Our broad search results were narrowed by excluding all studies concerned with HPAIV, human, swine or equine influenza, vaccines, or outbreaks in poultry (Fig 1). In order to be included in our analysis a study had to quantitatively assess the persistence of an LPAIV virus strain in water, and provide at least one environmental moderator variable of interest (see below).

Quantitative studies either measured the 50% tissue culture infective dose (TCID_50_) or 50% egg infective dose (EID_50_), determined by the dose of virus that causes a cytopathic effect in 50% of the inoculated tissue or eggs. A single study presented information as plaque forming units (PFU), for which we converted the values using a standard conversion of 1 TCID_50_ = 0.69 PFU[41]. TCID_50_, or EID_50_, was used to calculate the log-scale reduction in infective dose, Rt. Specifically, Rt is the time taken to achieve a 1-unit log-scale reduction in the TCID_50_ (or EID_50_) and is usually provided in days (although two studies presented Rt in minutes or months, and were subsequently converted to days), thus providing a measure of the degradation rate of the virus strain.

The initial search terms returned 7862 records (see Fig 1). We removed 4183 duplicates, leaving 3679 studies. A further 3456 studies were excluded at the first level of screening because they did not meet the inclusion criteria (see above), and a further 150 studies, which involved poultry, human, swine or other species, or were related to vaccination, immunology or treatment, were removed at the second screening. We assessed 73 full-text articles for eligibility, and excluded 45 studies because they did not specifically examine LPAIV in wildlife, LPAIV in the environment, or shedding of the virus. From this final set we excluded 25 studies because they were observational studies only with no quantitative information on persistence of the virus. The remaining 28 studies were included in the final systematic review, with two studies excluded from the quantitative synthesis as they did not contain empirical data that could be meaningfully extracted.

### Data collection

**Fig 2:**
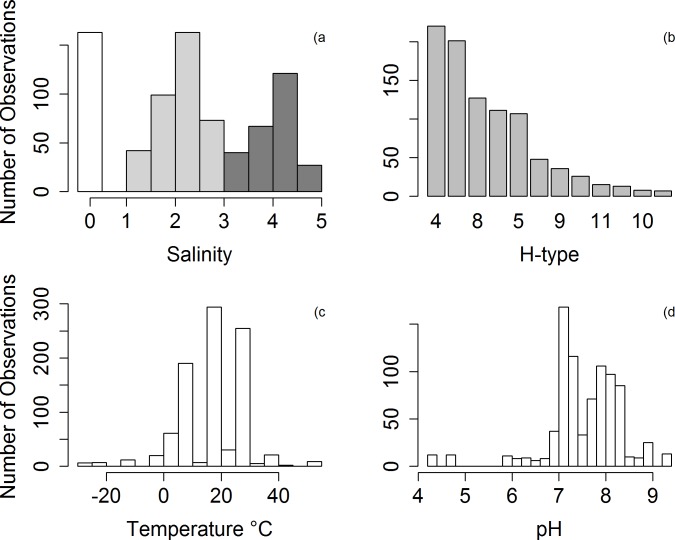
Distribution of observed values for the moderator variables across the full dataset: (A) log_10_ salinity; (white = 0ppm, grey = <1000ppm, black = ≥ 1000ppm); (B) H-type in descending order of observations; (C) temperature; and (D) pH. Plot (A) is a barplot, and plots (B), (C) and (D) are histograms.

We found (and included) 26 studies conducted between 1978 and 2014, which contained 1824 experimental outcomes. Due to the missing information, we were only able to estimate effect sizes (see below) for 919 (50.4%) individual outcomes of persistence (Table 1). For each individual outcome of persistence we extracted the following moderator variables:

1. temperature; reported in degrees celsius, (n = 919 data points, range -30 to 55°C);
2. pH; reported in standard units, (n = 836 data points, range 4.2–9.4);
3. salinity; converted to parts per million (ppm) across all studies (n = 795 data points, range 0 – 42477ppm). Where salinity was not reported directly, but reference was made to distilled water, salinity was assumed to be zero ppm. The distribution of salinities showed a clear tri-modal pattern (Fig 2A) and for further analyses they were grouped into three categories, salinity group 0 (0ppm; n = 163 data points), salinity group 1 (1 to 1000ppm; n = 307 data points) and salinity group 2 (>1000 ppm; n = 255 data points);
4. water type; categorised as sterilised, distilled, filtered or unfiltered based on descriptions within the text of each study. Water was classified as distilled when expressly described so in the study (n = 211 data points). Unfiltered water was assumed when no information was given for the nature of water, or when the water sample was expressly reported as being unfiltered (n = 104 data points). Filtered water was assigned to any experiments that described a filtration technique (n = 481 data points). Sterilised water refers to any mention of the use of an autoclave, regardless of whether or not it was filtered prior to sterilisation (n = 34 data points);
5. H-type; every study detailed the H and N type of the strain used (n = 919 data points; H1 = 13, H2, = 26, H3 = 201, H4 = 220, H5 = 107, H6 = 111, H7 = 48, H8 = 127, H9 = 36, H10 = 8, H11 = 15, H12 = 7).

**Table 1:** Papers meeting all inclusion criteria for quantitative analysis, including all experimental outcomes, the number of reported results and model fit statistics. The primary author, year of publication, and country the study was conducted and published in are provided. ‘H’ is the number of different H-types reported in the experiment(s), strains is the number of different strains (multiple version of one H-type might be used) in each study. Temperatures, pHs and salinities are the number of different levels recorded for each factor. Experimental combinations is the total number of temperature/pH/salinity/strain combinations that could be directly ascertained from the paper. Rt, R^2^ and slope is the number of individual reports of each result in each paper. The origin and year of isolation for all strains used in each study are available in S1 Table.

Study	Year	Country	H	Strains	Temperatures	pHs	Salinities	Experimental combinations	Rt	R^2^	Slope
Brown et al	2007	N. America	2	8	2	1	3	48	48	48	48
Brown et al	2009	N. America	12	12	3	5	5	900	60	0	0
Davidson et al	2010	Israel	1	3	3			7	7	7	7
Graiver et al	2009	N. America	1	1	3	2	4	8	8	0	8
Guan et al	2009	Canada	6	1	4	2		8	8	0	0
Harris et al	2010	N. America	2	2	2	2	2	12	10	0	0
Keeler et al	2012	N. America	2	2	3	15	15	90	90	90	90
Keeler et al	2013	N. America	2	11	1	1	1	27	27	0	0
Keeler et al	2014	N. America	3	3	3	38	38	342	342	0	0
Lebarbenchon et al	2011	N. America	2	2	5	3	2	18	18	18	0
Lebarbenchon et al	2012	N. America	3	5	5	1	1	25	25	0	0
Mihai et al	2011	Romania	1	1	3	3	3	27	9	9	9
Nazir et al	2010	Germany	3	3	5	3	3	45	45	45	45
Nazir et al	2010b	Germany	3	3	5	1	1	20	20	20	20
Nazir et al	2011	Germany	3	3	4			32	32	0	0
Negovetich and Webster	2010	N. America	1	7	2			3	0	0	21
Nielsen et al	2013	Denmark	2	2	3	1	3	35	16	0	0
Shoham et al	2012	Japan	2	2	2	3	3	12	12	12	12
Stallknecht et al	2010	N. America	2	4	1	1	1	4	4	4	4
Stallknecht et al	1990	N. America	3	3	2	8	7	95	22	22	22
Stallknecht et al	1990b	N. America	5	5	3	1	1	11	11	11	11
Terregino et al	2009	Italy	1	7	2			14		0	21
Webster et al	1978	N. America	1	1	2	2	2	4	4	4	4
Zarkov & Urumova	2013	Bulgaria	1	1	3	1	1	5	5	5	5
Zarkov	2006	Bulgaria	2	2	5	5	5	20	20	0	0
Zhang et al	2014	China	2	2	3	4	4	47	47	47	47
TOTALS								**1824**	**873**	**333**	**365**

From each study we extracted the following summary statistics; or a subset when the full information was unavailable:

1. Rt; estimated duration of infectivity (in days) of the virus strain, being the time taken to achieve a 1-unit log-scale reduction in the TCID_50_ (or EID_50_);
2. Regression slope and standard error; the estimated slope coefficient from a reported linear regression model between 50% infective dose (TCID_50_, or EID_50_) and time (days);
3. intercept; the constant dependent value from the reported linear regression model (see ii);
4. n; the number of experimental samples used to calculate Rt. Where *n* was not reported directly (2 out of 26 studies) we estimated it by multiplying the number of time points and the number of replicates, or counting the minimum number of time points for which we had clear evidence;
5. R^2^; Coefficient of Determination from the reported linear regression model (see ii).

### Statistical analysis

We have considered two measures of effect size, Rt (log-scale reduction in infective dose) and Zr (Fisher’s z-transformed correlation coefficient). Rt was extracted, where possible, directly from the empirical results of the published studies. Zr was calculated from the correlation coefficients (see below) by converting the regression model R^2^ values to correlation coefficients following equation (1) in Nakagawa [42]. All statistical analyses were conducted using the R software environment for statistical and graphical computing (v.3.1.0) [43].

Studies included in the meta-analysis did not always provide both an Rt and R^2^ value (with associated slope, standard error and intercept), and some studies only provided a plotted figure (bivariate scatterplot) of the association between log TCID_50_ (or EID_50_) and time. Where a figure was provided and the diagnostic information was unavailable we data-mined the figures using a Plot Digitizer [44]. The figures were then reconstructed in the R software environment for statistical and graphical computing [43] and a simple linear regression model was fitted, allowing us to estimate the values for Rt, R^2^, linear slope (and standard error), and model intercept, indirectly. The full dataset is available in online S3 Table.

We used the meta-analysis package *metafor* [45] to transform and visualise the effect sizes, for model fitting, and for the calculation of within study variance (i.e., effect size heterogeneity). Correlation coefficients were transformed to their Fisher’s *z*-transformed correlation coefficients (*Zr*), and their sampling variances calculated, using the *escalc* function in *metafor*. Where the sampling variances could not be calculated, due to small sample size (n ≤ 4), they were excluded from further analysis.

We used a modification of Egger’s regression test [46] to evaluate evidence for publication bias in our measure of the strength of the effect size of the persistence of LPAIV (Zr). The test was conducted by modifying the multi-level meta-regression models to include the square root of the sampling variance estimates associated with each effect size as an additional moderator variable. Where the intercept of the resulting model does not differ significantly from zero there is no evidence for publication bias. We did not apply the Egger regression test to our measure of the magnitude of the effect size (Rt) because the expected value of this effect size will always be greater than zero, given that persistence can only decrease with time, not increase, in the absence of transmission between hosts.

We followed a ‘meta-regression’ approach [47] to test the effects of multiple factors (including both continuous and categorical moderator variables) in a single model. We constructed a random-effects model to account for the random variation among studies (i.e., study ID was included as the among study random effect) and the non-independence of multiple data points from the same study (i.e., experiment within each study ID was included as the within study-level random effect). All moderator variables were included simultaneously as fixed effects in the model. All confidence intervals are 95% intervals. We refitted the meta-regression model using Bayesian estimation (using package R2JAGS) to extract the conditional between-study effect size estimates (and credible intervals) for plotting, as these were not available from the model output using metafor.

We evaluated the relative rankings of candidate models that included all possible subsets of the four predictor variable using an information-theoretic approach (AIC_c_ [48]) to determine the relative importance of each predictor. The sum of the Akaike weights from all models in which a predictor variable was included was used as its measure of relative importance. Relative importance was not calculated for the models of Rt because the model that included all four predictors had almost all the relative weight, so all variables would rank as having maximum importance.

We repeated our analysis on a subset of the data using only the specific H-types H3, H4 and H8 (n = 201, n = 220, n = 127, respectively), enabling us to test for differences in persistence between the three most commonly studied H types. We also compared the overall responses of the studies with those of the subset (of most commonly studied H-types), for confirmation of any observed patterns.

We conducted a contrast analysis for the meta-regression model to test for the influence of different levels of the moderator variables. When predicting the effects of a specific moderator variable the other moderators in the model were set to pre-determined reference values, based on a median temperature and pH of the available data, and baseline levels for water type and salinity. The reference (baseline) conditions, which we used to compare the effects of the individual moderators, were a temperature of 17°C, fresh, sterilised water and a pH of 7.6.

Further investigation of the effect of salinity, as a continuous variable, was conducted by removing the large number of laboratory-based 0ppm data points (n = 148).

The heterogeneity statistic, I^2^, was used to quantify the relative proportions of among-study variation, within-study variation, and measurement variation [49]. This is particularly important in meta-analysis as it provides an estimate of model consistency [50]. Simple (rule-of-thumb) summary thresholds for the interpretation of I^2^ are considered to fall into overlapping brackets; 0–40% low, 30–60% moderate, 50–90% substantial, and 75–100% considerable heterogeneity [51].

## Results

### Data Description

The quantitative meta-analysis included 26 studies that investigated the environmental variables affecting the persistence of LPAIV in water (Table 1). Just three temperatures (10, 17 and 28°C) accounted for 72% of all the individual temperatures studied (Fig 2B). Similarly, for pH 24% of the results were obtained from just two values (7.2 and 7.4; Fig 2D). Fresh water (Salinity of 0ppm) was associated with 17% of the extracted data. This non-uniform sampling distribution was also evident for H-type where H3 and H4 were the most frequently studied, accounting for 45.8% of all the observed data (Fig 2A).

**Fig 3:**
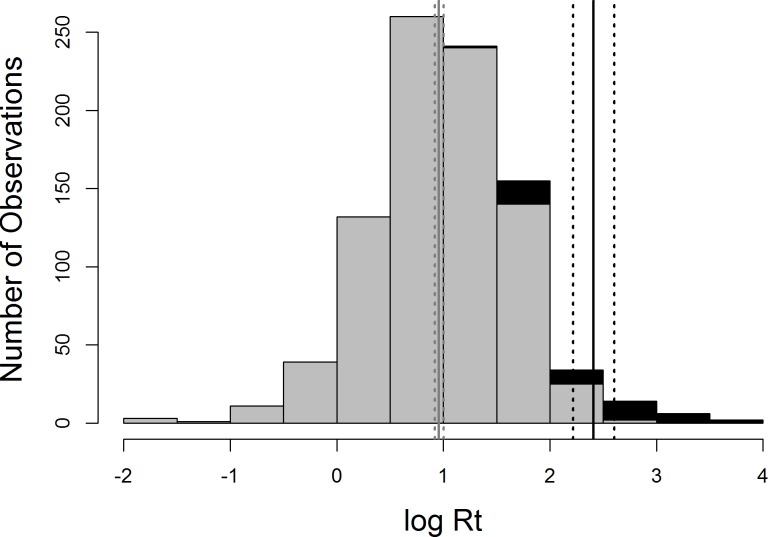
Distribution of log_10_Rt for the two temperature classes; non-frozen (dark grey, >4°C), and frozen (black, <4°C). The mean persistence of each group is given by the solid line, 95%confidence intervals are denoted by the dotted lines (non-frozen average = 22.88 days, S.D. = 40.46; frozen average = 691.59 days, S.D. = 1032.13)

There was a significant difference in persistence between the frozen (<4°C) and non-frozen (≥4°C) water temperatures (t = 4.4, df = 44, p < 0.001; Fig 3) with virus persisting for substantially longer (average = 691.6 days, s.e. = 158.21) in frozen compared to non-frozen samples (average = 22.9 days, s.d. = 40.5) (Fig 3). For all further analyses we only included samples with a temperature equal to or greater than 4°C; this temperature was the lowest temperature studied in liquid, rather than solid state, water.

The Egger’s regression rest for zero intercept was marginally significant (P = 0.041), providing support for the possibility of publication bias in this effect size measure.

### Meta-regression

#### Persistence (Rt)

**Fig 4:**
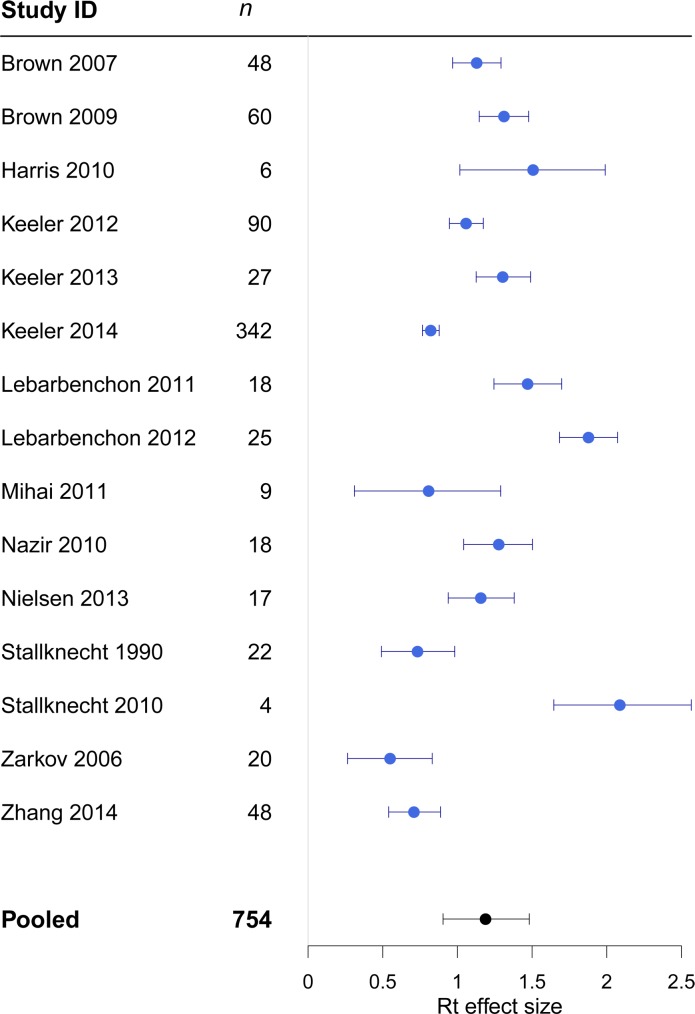
Forest plots showing heterogeneity between studies in the effect sizes for Rt. *n* = number of observations within each study; error bars show 95% credible intervals. The 'pooled' estimate shows the meta analytic effect size.

The overall effect size of the persistence of LPAIV in water was 1.2 (CI = 0.9–1.5). The average effect size varied substantially across studies (Fig 4). The largest variance was attributable to within study heterogeneity (I^2^_residual_ = 43.6%; variance = 0.21) (Table 2). The addition of all of the moderator variables (in a full model) considerably reduced the amount of the variance attributable to within-study differences in effects, and the largest component of variance was then attributable to between study differences (I^2^_study_ = 48.0%; variance = 0.110) (Table 2).

**Table 2:** The heterogeneity measure I^2^ (%) and variance for each model run with Rt and Zr accounted for by between study and within study variance, and measurement error. ID was included as the random effect, and the full model included moderator variables of temperature, pH, salinity and water type for the full dataset. The subset model included moderator variables of temperature, pH, salinity, water type and H-type for Rt, and temperature, pH, salinity and H-type for Zr.

		Between study (I^2^%, variance)	Within study (I^2^%, variance)	Measurement error (I^2^%, variance)
**Rt**	**Full dataset**			
	ID as random effect	41.9 (0.205)	43.6 (0.213)	14.4(0.071)
	Including moderator variables	48.0 (0.11)	21.4 (0.049)	30.6 (0.071)
	**Subset**			
	ID as random effect	34.3 (0.07)	33.3 (0.068)	32.4 (0.067)
	Including moderator variables	31.7 (0.061)	33.5 (0.064)	34.8 (0.067)
**Zr**	**Full dataset**			
	ID as random effect	27.8 (0.101)	42.8 (0.155)	29.5 (0.107)
	Including moderator variables	34.2 (0.12)	35.2 (0.123)	30.6 (0.107)
	**Subset**			
	ID as random effect	2.9 (0.01)	72.0 (0.247)	25.2 (0.086)
	Including moderator variables	0 (0)	70.2 (0.203)	29.8 (0.086)

**Fig 5:**
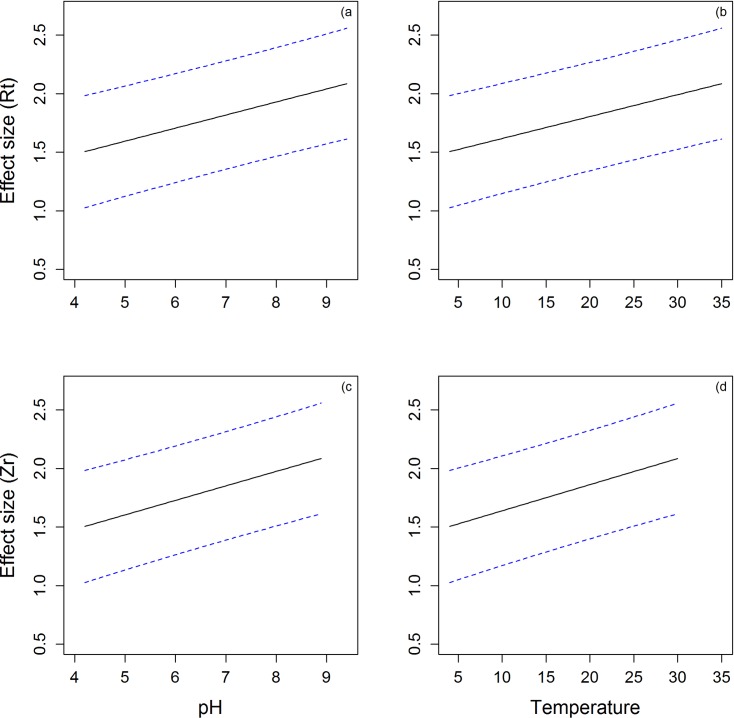
Predicted temperature and pH values from the contrasts model, with set priors of salinity and water type. Panels A and B present Rt for pH (A) and temperature (B). Panels C and D present fisher’s correlation coefficient, Zr for pH (C) and temperature (D).

**Fig 6:**
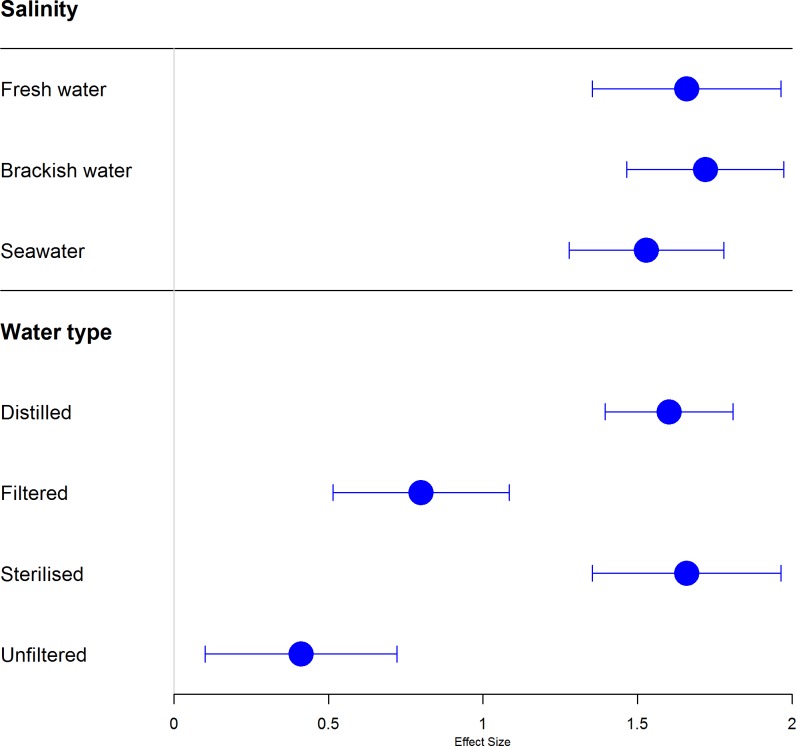
Meta-regression of the persistence of LPAIV using a mixed effects model. Salinity group and water type effect size displayed for each individual level when continuous variables are set to temperature of 17°C, pH 7.6. A smaller effect size indicates a more rapid degradation of the virus.

Moderator variables that explained heterogeneity in the full model included a positive effect of pH on persistence of LPAIV (Fig 5A), a negative effect of temperature (Fig 5B), and lower persistence in filtered and unfiltered water (compared with sterilised and distilled water) (Fig 6). A continuous measure of salinity (after removing samples where salinity was equal to 0ppm from the data set) was positively related to persistence (Table 3). In this model, a significant effect of pH was not detected, and all other moderator variables maintained similar effects to those in the model fitted to the full data set (Table 3).

**Table 3:** Estimated effect size (Rt) from model predictions for the full dataset and subset dataset, using salinity as a categorical, and then continuous, variable. Influential moderator variables are highlighted in bold.

	Model variables	Effect size (Rt)	95% CI for Rt	z-value	p-value
***Full dataset***					
	**pH**	**0.235**	**0.194, 0.275**	**11.36**	**<0.0001**
	**Temperature**	**-0.049**	**-0.052, -0.045**	**-27.12**	**<0.0001**
	Salinity Group 1	0.0637	-0.113, 0.239	0.70	0.485
	Salinity Group 2	-0.127	-0.289, 0.036	-1.52	0.128
	**Filtered water**	**-0.804**	**-1.036, -0.572**	**-6.79**	**<0.0001**
	**Unfiltered water**	**-1.186**	**-1.446, -0.926**	**-8.93**	**<0.0001**
	Sterilised water	0.056	-0.193, 0.306	0.443	0.658
***Salinity (>0 ppm) as a continuous variable***					
	pH	0.046	-0.050, 0.142	0.94	0.348
	**Temperature**	**-0.054**	**-0.058, -0.049**	**-21.55**	**<0.0001**
	**Salinity (log**_**10**_**)**	**-0.113**	**-0.154, -0.073**	**-5.51**	**<0.0001**
	**Filtered water**	**-1.361**	**-1.787, -0.936**	**-6.27**	**<0.0001**
	**Unfiltered water**	**-1.358**	**-1.888, -0.828**	**-0.90**	**<0.0001**
	Sterilised water	-0.183	-0.583, 0.217	-5.02	0.369
***H3*,*4*,*8 subset***					
	**pH**	**0.261**	**0.216, 0.306**	**11.40**	**<0.0001**
	**Temperature**	**-0.050**	**-0.054, -0.046**	**-22.35**	**<0.0001**
	Salinity Group 1	0.284	-0.411, 0.978	0.80	0.424
	Salinity Group 2	0.122	-0.568, 0.812	0.35	0.729
	Filtered water	-0.681	-1.403, 0.043	-1.84	0.065
	**Unfiltered water**	**-1.618**	**-2.367, -0.869**	**-0.50**	**<0.0001**
	Sterilised water	-0.217	-1.063, 0.629	-4.23	0.615
	H4	-0.028	-0.101, 0.046	-0.74	0.458
	**H8**	**-0.186**	**-0.274, -0.099**	**-4.16**	**<0.0001**
***Salinity (>0 ppm) as a continuous variable***					
	pH	0.018	-0.052, 0.088	0.50	0.619
	**Temperature**	**-0.056**	**-0.063, -0.050**	**-17.19**	**<0.0001**
	**Salinity (log**_**10**_**)**	**-0.128**	**-0.169, -0.086**	**-6.07**	**<0.0001**
	Filtered water	-0.750	-1.333, -0.168	-2.52	0.012
	Sterilised water	-0.232	-0.878, 0.413	-0.71	0.481
	H4	-0.046	-0.162, 0.070	-0.77	0.441
	H8	-0.123	-0.244, -0.002	-1.99	0.046

The overall effect size for the subset of common H-types (H3, H4, H8) was 1.3 (CI 1.1–1.5). The variance was evenly distributed between the three components: (i) between study; (ii) within study; and (iii) measurement error (Table 2). Although temperature, pH, unfiltered water and H8 were associated with persistence of LPAIV (Table 3), they explained only small amounts of the heterogeneity observed between and within studies (Table 2).

#### Fisher’s transformed correlation coefficient (Zr)

**Fig 7:**
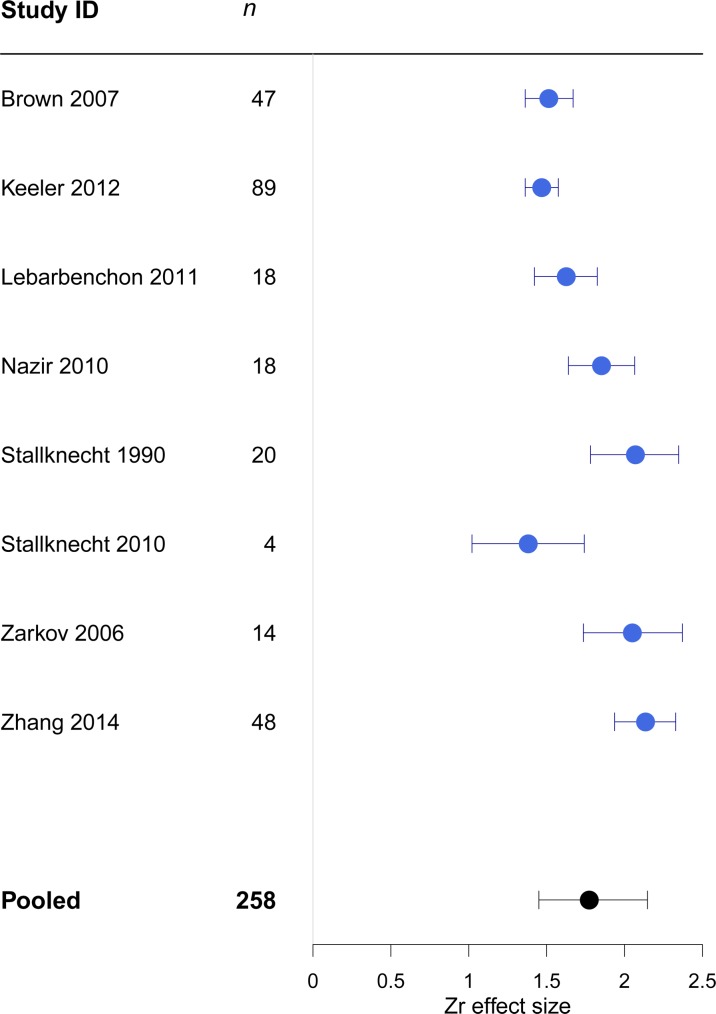
Forest plot showing heterogeneity between studies in the effect sizes for Zr. *n* = number of observations within each study; error bars show 95% credible intervals. The 'pooled' estimate shows the meta-analytic effect size.

The overall estimate for the strength of the effect size was 1.77 (CI 1.45, 2.14). The average effect size did not vary substantially across studies (Fig 7). The largest variance component was attributable to the within study variance (I^2^_residual_ = 42.8%). Only a small amount of the heterogeneity was accounted for by the moderator variables (Table 2).

Moderator variables that positively influenced persistence were warmer temperatures (Fig 5A), and higher salinities (>1000ppm) (Table 4). Both of these predictor variables were highly influential across all possible subsets of the full model (S2 Table). When the model was re-run with salinity as a continuous variable, without observations for 0ppm, the effect of salinity was no longer evident (Table 4).

**Table 4:** Estimated effect size (Zr) from model predictions for the full dataset and subset, using salinity as a categorical, and then continuous variable. Influential moderator variables are highlighted in bold.

	Model variables	Effect size (Zr)	95% CI for Zr	z-value	p-value
***Full dataset***					
	pH	0.027	-0.044, 0.098	0.74	0.461
	**Temperature**	**0.022**	**0.014, 0.031**	**5.17**	**<0.0001**
	Salinity Group 1	0.323	-0.043, 0.689	1.73	0.083
	Salinity Group 2	0.372	0.085, 0.570	2.54	0.011
	Filtered water	0.138	-0.472, 0.749	0.44	0.657
	Unfiltered water	0.069	-0.577, 0.715	-0.29	0.834
	Sterilised water	-0.060	-0.462, 0.342	0.21	0.771
***Salinity (>0 ppm) as a continuous variable***					
	pH	0.125	-0.032, 0.281	1.56	0.118
	Temperature	0.026	0.010, 0.042	3.24	0.001
	Salinity (log10)	0.133	-0.096, 0.362	1.14	0.256
	Filtered water	0.497	-0.103, 1.097	1.62	0.104
	Unfiltered water	0.270	-0.020, 1.055	0.75	0.452
	Sterilised water	0.517	-0.433, 0.974	1.89	0.059
***H3*,*4*,*8 subset***					
	pH	0.038	-0.510, 0.127	0.83	0.404
	**Temperature**	**0.028**	**0.015, 0.042**	**4.15**	**<0.0001**
	Salinity group 1	-0.137	-0.394, 0.120	-1.04	0.297
	Salinity group 2	0.098	-0.263, 0.458	0.53	0.596
	H4	-0.135	-0.338, 0.068	-1.30	0.193

Due to the smaller dataset for Zr (n = 302), we removed water type from the full model (see previous Results). The overall effect size for the subset of common H-types (H3, H4 and H8) was 1.5 (CI 1.3 1.7). The between study heterogeneity estimate (I^2^_study_) was small (2.9%; variance = 0.010), due to the small number of studies (and observations) retained in the subset; for within study and measurement error variances see Table 2. Temperature was the only variable with a notable effect in the model (Table 4), and, the explanation of heterogeneity in the model was not improved by including the moderator variables (Table 2).

## Discussion

With any emerging disease it is fundamental that we develop a comprehensive understanding of the consequences of interplay between the host, the agent, and the environment [52]. This conceptual and quantitative understanding will help to ensure greater surveillance efficacy, as well as prevention of future outbreaks and more accurate prediction and prevention of pandemics [17, 53]. Influenza A virus is a disease of pandemic potential, with multiple host species and a rapidly mutating genome [54]. LPAIV naturally circulates in waterbird hosts, and may often include an environmental component within its transmission dynamics [17, 19, 26, 28, 55]. The aquatic environment provides physical, chemical and biological challenges for LPAIV to overcome to ensure infectivity to a new recipient host [56].

Water type had a strong effect on the persistence of LPAIV, with unfiltered and filtered water significantly decreasing persistence (see also [32, 33, 36, 57, 58]). While the exact mechanism for the reduced persistence is not yet fully understood, Nazir et al (2010) suggested that virus particles may be both consumed by microbes, or adhere to particulate matter and no longer be infective, or become less infective, in more biologically active water. The biological content of water, including filter feeders and other invertebrates, has been found to have an effect on the inactivation of echoviruses [59], polioviruses and coxsackieviruses [60].

The temperatures a virus can withstand are crucial to their persistence, whether inside a host or freely surviving in the environment [1], and a trade-off between persistence at low environmental temperatures and the ability to endure higher temperatures in avian hosts has even been proposed [55]. Previous studies, as well as this study, have provided considerable insight into how LPAIV persistence and temperature are related, however, temperatures in natural environments rarely maintain a single steady level. Locations may have widely varying temperatures throughout one single 24 hour period; e.g., rivers and shallow lakes that can observe a 10°C change between day and night in the surface temperature [61]. Though we found temperature to have a strong consistent influence on persistence of the virus, the majority of available data is centred on just three temperatures, which do not adequately represent conditions in large areas of the world. Researchers need to examine the local habitat differences that can affect the variability in water temperature, and subsequently persistence of the virus, as well as continue to expand the range of temperatures studied to allow full characterisation of the response.

We found salinity to be an influential continuous variable, but not when it was grouped as a categorical variable (i.e., including laboratory grade fresh water 0ppm). The inconsistent response to salinity, even between viruses of the same H and N types, has been observed previously [24]. The more rapid degradation of virus in salt water, relative to ‘fresh’ water, is most likely due to structural changes within the virus in the presence of higher salt concentrations that affect the conformation of the nucleocapsid segments [62].

Persistence of LPAIV was negatively associated with the acidity of the water sample and it has been suggested that LPAIV remains infective for the longest time between pH of 7.2 and 8.4 [24, 63]. Viral fusion activity relies on pH to allow infection of a cell, with the haemagglutinin protein of the influenza virion experiencing a conformational change at low pH values that allows entry into the host cell [64]. Thus, the changes in the surface protein may go some way to explaining the more rapid loss of infectivity, and hence reduced persistence of LPAIV in low pH water.

Phenotypic diversity in response to temperature and pH have been suggested between individual viruses [24], and differences between strains at low temperatures have been proposed [17, 63, 65], but such differences have not yet been fully explored. Some differences between H-types have been noted under experimental conditions with different water types at low temperatures, however the difference is reduced when using unfiltered water [33]. We found strain-related differences for H8 compared to H3 when using a smaller dataset, however, we do not propose a mechanism for this difference as yet. Studies have suggested that viral genome composition has limited effects on virus persistence [66], and possibly no fitness cost to the wild bird populations [67]. Whilst there may be no fitness cost to the host, there may still be an evolutionary advantage to the virus, if different strains are able to persist in different environments (e.g. temperatures and pH), a relationship that warrants further exploration.

Naming convention for LPAIV includes the species that the strain was first isolated from, but there is not known to be an association with specific host species for individual strains. Alternatively, if there are differences between H-types with respect to their persistence under environmental conditions, they may be more likely to infect some hosts than others due to the individual host ecology. The investigation of inter-strain differences across a wider range of H-types, under different naturalistic conditions, would be beneficial before we can rule out any differences between strains that may affect persistence in the aquatic environment.

Quantitative meta-analysis, using multiple studies, can provide important synthesis and agreement across replicate experiments, and provides the best evidence for cause-effect relationships [68]. By employing meta-analysis methods we can make predictions across a wide range of environmental conditions. These approaches also ensure the conclusions drawn are robust and markedly reducing Type II errors [69]. Meta-regression analysis has allowed us to investigate the effect of the environmental variables on infectivity of LPAIV in water, which may be accounting for the substantial heterogeneity in the dataset [70]. Unfortunately, despite reporting 1824 experimental outcomes, we were only able to estimate an effect size for half of these experiments (50.4%; n = 919 data points); because of the very poor reporting of individual results, and test statistics, across these studies.

Future reporting of studies should include the following minimum information: (i) Rt, and the method by which it was calculated (38.0% did not report Rt or an equivalent); (ii) the sample size (i.e., the number of time points used, and the number of replicate experiments performed), given explicitly in only two studies; (iii) reproducible descriptions, and definitions of all of the variables including water type, (provided by 50.0% studies); and (iv), where linear models are fitted to the data, the R^2^ (given in all but one study where fitted) and standard errors of the slope estimate (reporting of the standard error of the slope was very limited). A comprehensive description of methods, including calculations, and transparency of results will allow comparison of studies and assimilation of results to provide a wider basis for further analysis and translation of effects.

Environmental variables have been previously sampled across a very limited (or unrepresentative) range of values. Temperatures included in the studies have mostly reflected the average summer and winter temperatures of the North American breeding grounds of the natural waterbird hosts (28 and 17°C) [24, 63, 71, 72]. Other areas of the world are subject to very different temperature ranges, with some areas of the globe regularly reading in the high 30s (e.g., our own part of South Australia, Adelaide), or having large variations between overnight and daytime temperatures.

A very small number of possible H-types (H3, H4, H8) accounted for more than five hundred data points (59.6%) in our meta-analysis, and some strains were included in multiple studies (see S1 Table). It is unclear why these H-types have been the most utilised, but most likely it is representative of a geographic and taxonomic bias in field sampling, by a relatively small number of researchers. It may also reflect a bias in the availability of stock virus for experiments, as the same stock virus used was for all four studies that examined the viral persistence of the H8 virus, and a similar situation is true of the H3 and H4 types. In any case, the same degree of coverage needs to be achieved for all H-types to ensure we can convincingly conclude whether (or not) there are any H-type related differences in persistence.

The majority of studies were conducted at neutral or near-neutral pH levels, 7.2 and 7.4, providing a good baseline, but providing little (or no) information on more acidic, (e.g. coastal lakes, pH ~5) or more alkaline waters (e.g. sea water, pH ~8.2) [73]. As climates change around the world, and hosts alter their migration patterns, we are likely to detect hosts in new areas shedding virus into a variety of novel aquatic environments, as well as experiencing more acidic and higher salinity water in the traditional breeding locations [39]. We recommend that studying a wider range of temperatures, pH and salinity, as well as H-types, would undoubtedly be informative.

While we found some support for the possibility of publication bias in our measure of the strength of the effect size of the persistence of LPAIV (Zr), we are willing to interpret this with caution. Our analysis included a large number of individual estimates across a reasonably small number of studies, with a high level of between- and within-study heterogeneity. However, we were not able to include half of these published results in study, because of poor reporting statistics and effect size estimates. While it is clear that more studies need to be conducted to address the poor coverage of environmental variables, and resulting knowledge gaps, we are not convinced that this means there is a substantial publication bias (or ‘file-drawer’ problem).

All of the experimental studies, included in our quantitative meta-analysis, were conducted under laboratory conditions. Without environmental realism, there are limitations to the applicability of the information gained from these experiments [17, 19]. This is particularly true for the large number of baseline studies of distilled water (0ppm) (17.7%), conducted at a static pH and temperature. Such conditions are rarely, if ever, found in ex-situ systems, and as yet there have been very few published reports of experiments that have explicitly accounted for daily environmental variations, or fluctuations. Studies included here used static states for pH, temperature and salinity, with the exception of those examining freeze-thaw degradation of the virus and one author [65, 66] who has begun the process of examining diurnal temperature variations; though the findings were not consistent, demonstrating that diurnally varying conditions need much more investigation.

Diurnal variations in temperature, water flow rate and depth, ultraviolet light (UV) exposure, turbidity, and biological diversity are just some of the environmental variables that we suggest need to be considered in future work. Water flow rates, through areas where waterbirds are shedding virus via their faeces, are likely to have a dilution effect, reducing the number of infective particles available for ingestion by the next host, in a given area of water. In water treatment plants, and numerous other applications, UV light is used to disinfect physical surfaces and water. Viruses can be particularly resistant to UV [74], but the amount of exposure required to affect the persistence of LPAIV in water to date has not been investigated; although there has been some work on the human H7N9 where more than 30 minutes exposure to UV within 75cm of the light source caused the death of the virus [75]. Recently, researchers have included ‘natural’ water in infectivity experiments, i.e. water samples taken from natural water bodies [32, 33, 65]. However, these experiments were all maintained at single (static) temperatures, and whilst the physicochemical properties of the water are reported from *in situ* measurements, there was minimal reporting of the final ‘laboratory’ values (2 of 10 studies provided final values).

Whilst abiotic factors such as temperature and salinity have a role to play in the persistence of virus, they are only a fraction of the whole story. Biotic factors including filter feeders and invertebrates need to be considered when attempting to understand the role of the natural environment [76]. Investigation into the bioaccumulation and/or inactivation of AIV by filter feeders and invertebrates has garnered interest in the last few years, with experiments using zebra mussels (*Dreissena polymorpha*) [77], freshwater Asiatic clams (*Corbicula fluminea*) [78] and water fleas (*Daphnia magna*) [79]. The results of the studies have been varied, with some providing evidence for bioaccumulation in the tissues of species which are a possible food source for waterbirds [77, 78], and others showing removal and inactivation of AIV by invertebrate communities [79].

Throughout this paper we have highlighted the need for environmental realism, and in part this can be achieved through the use of meta-analysis to assimilate all available information allowing the extrapolation of expected results for a given set of circumstances. A further step forward would be the construction of mesocosms, with water quality parameters in line with the conditions found in the wild. Although mesocosms can only mimic the natural environment, and will have constraints which limit the overall realism that can be achieved, the ability to allow for biological content and broader (fluctuating) physicochemical conditions will be an advancement in the field.

The role that invertebrates, which share waterbodies with waterbirds, play in the maintenance and transmission of AIV in the environment needs greater investigation, and could be a substantial step towards understanding the interactions that occur between biotic and abiotic variables [76]. Subsequently, combining mesocosm studies with those involving invertebrates will take us much closer to an overall understanding of the persistence and transmission of AIV in the aquatic environment.

There are multiple factors which have an influence on the persistence of viruses in the environment. Though we have focused on LPAIV, we have also described a methodology for health researchers and practitioners to apply meta-analytic techniques to wildlife diseases. We believe that these methods will continue to be particularly important when considering emerging diseases moving into new environments, or under anthropogenic environmental changes. Meta-analysis allows the consideration of the relationships among multiple variables, as well as determining the limitations of the sampling coverage to date. Some notable outbreaks and emergences in new areas, which may be ripe for meta-analysis include white nose syndrome in chiroptera [80, 81], Zika virus [82] and Ebola virus [83].

## Conclusions

Environmental variables clearly impact the persistence of LPAIV in water, and although the current range of moderator variables studied is limited, some important conclusions can be drawn. Water type and temperature have significant effects on the persistence of the virus, with colder temperatures allowing for greater persistence in the environment and unfiltered water reducing infectivity. Salinity was shown to have a significant effect on the persistence of the virus. In addition, pH has an effect on infectivity, although the relationship is less clear when investigated in association with salinity. Our study has highlighted that a small measured range, in a limited number of variables, accounts for the majority of research effort to date. We greatly hope that future experimental studies will continue to investigate outside these ranges. This is of particular importance for studies conducted outside the geographical range of past research (i.e., North America and Europe) where the range of conditions may be wider, the environment more variable, and the hosts following different life-histories from that of the Northern hemisphere. We also hope that there will be a further shift towards environmental realism through the use of mesocosms and the integration of invertebrate accumulation and inactivation studies, and that eventually *in situ* experiments may be possible.

## Supporting Information

S1 Fig PRISMA 2009 checklist. (DOC)

S1 Table Study, H-type, N-type, year of isolation, species of isolation, Country and State (where applicable) of isolation, and designation of each viral subtype used in all included studies. (DOCX)

S2 Table Model selection table for Zr using showing model structure, AICc and weights for each model, accounting for combinations of the four moderator variables included in the full model. (DOCX)

S3 Table The full dataset of extracted standardised data. (CSV)
