# Correction: Persistence of Low Pathogenic Influenza A Virus in Water: A Systematic Review and Quantitative Meta-Analysis

**DOI:** 10.1371/journal.pone.0168789

**Published:** 2016-12-16

**Authors:** Antonia E. Dalziel, Steven Delean, Sarah Heinrich, Phillip Cassey

Fig 5 is incorrect. The authors have provided a corrected version here.

**Fig 5:**
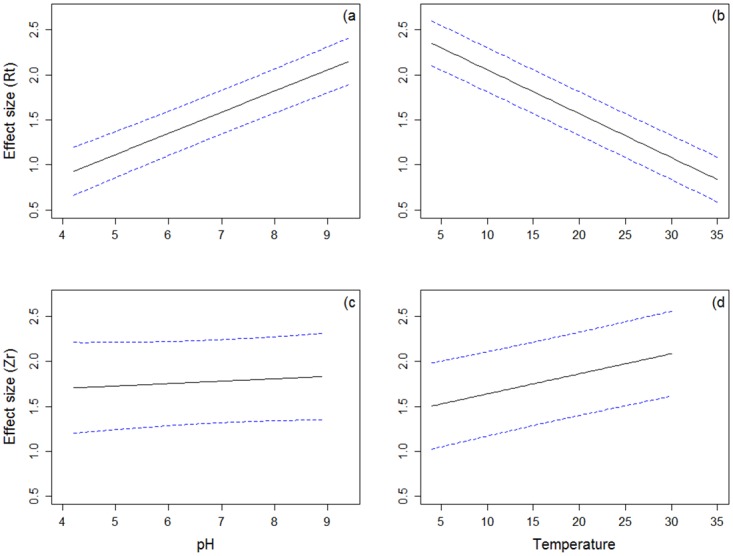
Predicted temperature and pH values from the contrasts model, with set priors of salinity and water type. Panels A and B present Rt for pH (A) and temperature (B). Panels C and D present fisher’s correlation coefficient, Zr for pH (C) and temperature (D).
