# Supplementary material for: Persistence of Low Pathogenic Influenza A Virus in Water: A Systematic Review and Quantitative Meta-Analysis
Source: PLoS One. 2016 Oct 13;11(10):e0161929. doi: 10.1371/journal.pone.0161929 (PMC5063340; doi:10.1371/journal.pone.0161929)
Supplement: S1 Table — (DOCX) [file pone.0161929.s002.docx]

| **Study [Reference]** | **H type** | **N type** | **Year of strain isolation** | **Isolated from** | **Country isolated** | **State/Region** | **Designation** |
| --- | --- | --- | --- | --- | --- | --- | --- |
| Brown et al [24] | 2 | 4 | 2001 | Mallard | United States | Texas | A/Blue-winged Teal/TX/421717/2001 (H2N4) |
| Brown et al [24] | 11 | 6 | 2000 | Dunlin | United States | Delaware | A/Dunlin/DE/AI00-1459/2000 (H11N6) |
| Brown et al [24] | 1 | 1 | 1987 | Green-winged Teal | United States | Louisiana | A/Green-winged Teal/LA/213GW/1987 |
| Brown et al [24] | 3 | 2 | 1999 | Mallard | United States | Minnesota | A/Mallard/MN/199036/1999 (H3N2) |
| Brown et al [24] | 4 | 6 | 1999 | Mallard | United States | Minnesota | A/Mallard/MN/199057/1999 (H4N6) |
| Brown et al [24] | 5 | 2 | 2000 | Mallard | United States | Minnesota | A/Mallard/MN/346250/2000 (H5N2) |
| Brown et al [24] | 12 | 5 | 2000 | Mallard | United States | Delaware | A/Mallard/MN/355788/2000 (H12N5) |
| Brown et al [24] | 8 | 4 | 2001 | Northern Pintail | United States | Texas | A/Northern Pintail/TX/421716/2001 (H8N4) |
| Brown et al [24] | 7 | 6 | 2005 | Northern Shoveler | United States | North Carolina | A/Northern Shoveler/NC/1523546/2005 (H7N6) |
| Brown et al [24] | 10 | 7 | 2000 | Red Knot | United States | Delaware | A/Red Knot/DE/AI00-1329/2000 (H10N7) |
| Brown et al [24] | 6 | 4 | 2001 | Ring-billed Gull | United States | Georgia | A/Ring-billed Gull/GA/421733/2001 (H6N4) |
| Brown et al [24] | 9 | 2 | 2003 | Ruddy Turnstone | United States | New Jersey | A/ruddy turnstone/NJ/1916409/2003 (H9N2) |
| Brown et al [42] | 7 | 4 | 2002 | Blue-winged Teal | United States | Texas | A/Blue-winged Teal/TX/578597/02 (H7N4) |
| Brown et al [42] | 7 | 3 | 2000 | Laughing Gull | United States | Delaware | A/Laughing Gull/DE/AI00-2455 (H7N3) |
| Brown et al [42] | 5 | 2 | 1998 | Mallard | United States | Minnesota | A/Mallard/MN/182742/98 (H5N2) |
| Brown et al [42] | 7 | 3 | 1998 | Mallard | United States | Minnesota | A/Mallard/MN/182761/98 (H7N3) |
| Brown et al [42] | 5 | 3 | 2000 | Mallard | United States | Minnesota | A/Mallard/MN/355790/00 (H5N3) |
| Brown et al [42] | 7 | 3 | 2002 | Ruddy Turnstone | United States | Delaware | A/Ruddy Turnstone/DE/650635/02 (H7N3) |
| Brown et al [42] | 5 | 7 | 2001 | Ruddy Turnstone | United States | New Jersey | A/Ruddy Turnstone/NJ/828219/01 (H5N7) |
| Brown et al [42] | 5 | 8 | 2001 | Ruddy Turnstone | United States | New Jersey | A/Ruddy Turnstone/NJ/828227/01 (H5N8) |
| Davidson et al [43] | 9 | 2 | 2006 | Chicken | Israel | Gshor | A/Ck/Gshor/1525/10/12/06 |
| Davidson et al [43] | 9 | 2 | 2002 | Turkey | Israel | Givat Haim | A/Ty/Givat Haim/965/17/03/02 |
| Davidson et al [43] | 9 | 2 | 2004 | Turkey | Israel | Shadmot Dvora | A/Ty/Shadmot Dvora/1567/06/01/04 |
| Graiver et al [44] | 6 | 2 | 2001 | Chicken | United States | California | A/Ck/CA/101247/01 (H6N2) |
| Guan et al [45] | 6 | 2 | 1965 | Chicken | United States | Massachusetts | A/Tky/MA/3740/65 (H6N2) |
| Harris et al [46] | 3 | 2 | 1999 | Mallard | United States | Minnesota | A/Mallard/MN/199036/99 (H3N2) |
| Harris et al [46] | 8 | 4 | 2001 | Northern Pintail | United States | Texas | A/Northern Pintail/TX/421716/2001 (H8N4) |
| Keeler et al [32] | 3 | 2 | 1999 | Mallard | United States | Minnesota | A/Mallard/MN/199036/99 (H3N2) |
| Keeler et al [32] | 4 | 6 | 1999 | Mallard | United States | Minnesota | A/Mallard/MN/199057/99 (H4N6) |
| Keeler et al [33] | 4 | 6 | 1999 | Mallard | United States | Minnesota | A/Mallard/MN/199044/1999 (H4N6) |
| Keeler et al [33] | 3 | 8 | 1999 | Mallard | United States | Minnesota | A/Mallard/MN/199106/1999 (H4N6) |
| Keeler et al [33] | 3 | 8 | 2007 | Mallard | United States | Minnesota | A/Mallard/MN/Sg-00048/2007 (H3N8) |
| Keeler et al [33] | 4 | 6 | 2007 | Mallard | United States | Minnesota | A/Mallard/MN/Sg-00050/2007 (H4N6) |
| Keeler et al [33] | 3 | 8 | 2007 | Mallard | United States | Minnesota | A/Mallard/MN/Sg-00051/2007 (H3N8) |
| Keeler et al [33] | 4 | 6 | 2007 | Mallard | United States | Minnesota | A/Mallard/MN/Sg-00053/2007 (H4N6) |
| Keeler et al [33] | 4 | 6 | 2007 | Mallard | United States | Minnesota | A/Mallard/MN/Sg-00063/2007 (H4N6) |
| Keeler et al [33] | 3 | 8 | 2007 | Mallard | United States | Minnesota | A/Mallard/MN/Sg-00167/2007 (H3N8) |
| Keeler et al [33] | 8 | 4 | 2001 | Northern Pintail | United States | Texas | A/Northern Pintail/TX/421716/01 (H8N4) |
| Keeler et al [35] | 3 | 2 | 1999 | Mallard | United States | Minnesota | A/Mallard/MN/199036/99 (H3N2) |
| Keeler et al [35] | 4 | 6 | 1999 | Mallard | United States | Minnesota | A/Mallard/MN/199057/99 (H4N6) |
| Keeler et al [35] | 8 | 4 | 2001 | Northern Pintail | United States | Minnesota | A/Northern Pintail/TX/421716/01 (H8N4) |
| Lebarbenchon et al [47] | 3 | 6 | 2006 | Surface Water | United States | Minnesota | A/Surface water/MN/NW1-T/2006 (H4N6) |
| Lebarbenchon et al [47] | 3 | 8 | 2007 | Surface Water | United States | Minnesota | A/Surface water/MN/W07-2241/2007 (H3N8) |
| Lebarbenchon et al [48] | 6 | 8 | 2007 | Green-winged Teal | United States | Minnesota | A/Green-winged teal/MN/Sg-00197/2007 (H6N8) |

| Lebarbenchon et al [48] | 6 | 2 | 2007 | Mallard | United States | Minnesota | A/Mallard/MN/Sg-00107/2007 (H6N2) |
| --- | --- | --- | --- | --- | --- | --- | --- |
| Lebarbenchon et al [48] | 3 | 8 | 2007 | Mallard | United States | Minnesota | A/Mallard/MN/Sg-00169/2007 (H3N8) |
| Lebarbenchon et al [48] | 6 | 1 | 2007 | Mallard | United States | Minnesota | A/Mallard/MN/Sg-00170/2007 (H6N1) |
| Lebarbenchon et al [48] | 4 | 8 | 2007 | Mallard | United States | Minnesota | A/Mallard/MN/Sg-00219/2007 (H4N8) |
| Mihai et al [49] | 5 | 1 | 2004 | - | Vietnam | - | A/Viet Nam/1194/2004 |
| Nazir et al [50] | 4 | 6 | 2003 | Mallard | Germany | - | A/Mallard/Germany/Wv1732-34/03 (H4N6) |
| Nazir et al [50] | 6 | 8 | 2007 | Mute Swan | Germany | - | A/Mute Swan/Germany/R2927/07 (H6N8) |
| Nazir et al [50] | 5 | 1 | 2005 | Teal | Germany | - | A/Teal/Germany/Wv632/05 (H5N1) |
| Nazir et al [51] | 4 | 6 | 2003 | Mallard | Germany | - | A/Mallard/Germany/Wv1732-34/03 (H4N6) |
| Nazir et al [51] | 6 | 8 | 2007 | Mute Swan | Germany | - | A/Mute Swan/Germany/R2927/07 (H6N8) |
| Nazir et al [51] | 5 | 1 | 2005 | Teal | Germany | - | A/Teal/Germany/Wv632/05 (H5N1) |
| Nazir et al [52] | 4 | 6 | 2003 | Mallard | Germany | - | A/Mallard/Germany/Wv1732-34/03 (H4N6) |
| Nazir et al [52] | 6 | 8 | 2007 | Mute Swan | Germany | - | A/Mute Swan/Germany/R2927/07 (H6N8) |
| Nazir et al [52] | 5 | 1 | 2005 | Teal | Germany | - | A/Teal/Germany/Wv632/05 (H5N1) |
| Negovetich and Webster [53] | 2 | 3 | 1996 | Mallard | Canada | Alberta | A/Mallard/Alberta/201/1996 (P2E1) |
| Negovetich and Webster [53] | 2 | 3 | 1998 | Mallard | Canada | Alberta | A/Mallard/Alberta/205/1998 (P2E1) |
| Negovetich and Webster [53] | 2 | 3 | 1998 | Mallard | Canada | Alberta | A/Mallard/Alberta/226/1998 (E4) |
| Negovetich and Webster [53] | 2 | 3 | 1988 | Mallard | Canada | Alberta | A/Mallard/Alberta/353/1988 (P2E1) |
| Negovetich and Webster [53] | 2 | 3 | 1985 | Mallard | Canada | Alberta | A/Mallard/Alberta/376/1985 (P1E1) |
| Negovetich and Webster [53] | 2 | 3 | 1977 | Mallard | Canada | Alberta | A/Mallard/Alberta/77/1977 (P2E1) |
| Negovetich and Webster [53] | 2 | 3 | 2003 | Mallard | Canada | Alberta | A/Mallard/Alberta/79/2003 (E2) |
| Nielsen et al [54] | 7 | 1 | 2008 | Mallard | Denmark | - | A/Mallard/Denmark/52-351/08 (H7N1) |
| Nielsen et al [54] | 5 | 7 | 2003 | Mallard | Denmark | - | A/Mallard/Denmark/64.650/03 (H5N7) |
| Shoham et al [55] | 5 | 2 | 2006 | Northern Pintail | Japan | Akita | A/Northern Pintail/Akita/714/06 (H5N2) |
| Shoham et al [55] | 7 | 1 | 2004 | Northern Pintail | Japan | Aomori | A/Northern Pintail/Aomori/395/04 (H7N1) |
| Stallknecht et al [17] | 7 | 3 | 2001 | Laughing Gull | United States | New York | A/Laughing Gull/NY/A100-2455/2001 (H7N3) |
| Stallknecht et al [17] | 7 | 3 | 1998 | Mallard | United States | Minnesota | A/Mallard/MN/182761/1998 (H7N3) |
| Stallknecht et al [17] | 5 | 3 | 2000 | Mallard | United States | Minnesota | A/Mallard/MN/355790/2000 (H5N3) |
| Stallknecht et al [17] | 5 | 7 | 2001 | Ruddy Turnstone | United States | New Jersey | A/Ruddy Turnstone/NJ/828219/2001 (H5N7) |
| Stallknecht et al [56] | 4 | 6 | 1987 | Blue-winged Teal | United States | Louisiana | A/Blue-winged Teal/LA/44B/87 (H4N6) |
| Stallknecht et al [56] | 10 | 7 | 1988 | Green-winged Teal | United States | Louisiana | A/Green-winged Teal/LA/169GW/88 (H10N7) |
| Stallknecht et al [56] | 6 | 2 | 1987 | Mottled Duck | United States | Louisiana | A/Mottled Duck/LA/38M/87 (H6N2) |
| Stallknecht et al [57] | 12 | 5 | 1987 | Blue-winged Teal | United States | Louisiana | A/Blue-winged Teal/LA/188B/87 (H12N5) |
| Stallknecht et al [57] | 4 | 6 | 1987 | Blue-winged Teal | United States | Louisiana | A/Blue-winged Teal/LA/44B/87 (H4N6) |
| Stallknecht et al [57] | 3 | 8 | 1987 | Gadwall | United States | Louisiana | A/Gadwall/LA/17G/87 (H3N8) |
| Stallknecht et al [57] | 10 | 7 | 1988 | Green-winged Teal | United States | Louisiana | A/Green-winged Teal/LA/169GW/88 (H10N7) |
| Stallknecht et al [57] | 6 | 2 | 1987 | Mottled Duck | United States | Louisiana | A/Mottled Duck/LA/38M/87 (H6N2) |
| Terregino et al [58] | 7 | 1 | 1999 | Turkey | Italy | - | A/Turkey/Italy/3675/99 (H7N1) |
| Terregino et al [58] | 7 | 3 | 2003 | Turkey | Italy | - | A/Turkey/Italy/4608/03 (H7N3) |
| Webster et al [30] | 3 | 6 | 1974 | Duck | United States | Tennessee | A/Duck/Memphis/546/74 (H3N6) |
| Zarkov & Urumova [59] | 11 | 6 | 1956 | Duck | England | - | A/Duck/England/56 (H11N6) |
| Zarkov & Urumova [59] | 6 | 2 | 2005 | Mallard | Bulgaria | - | Not supplied |
| Zarkov [31] | 6 | 2 | 2005 | Mallard | Bulgaria | - | Not supplied |
| Zhang et al [34] | 5 | 1 | 2004 | - | China | Henan | A/Henan/12/2004(H5N1) |
| Zhang et al [34] | 9 | 2 | 2002 | - | China | Jiangsu | A/Jiangsu/11/2002(H9N2) |

**Table S1: Study, H-type, N-type, year of isolation, species of isolation, Country and State (where applicable) of isolation, and designation of each viral subtype used in all included studies.**
