# Supplementary material for: Persistence of Low Pathogenic Influenza A Virus in Water: A Systematic Review and Quantitative Meta-Analysis
Source: PLoS One. 2016 Oct 13;11(10):e0161929. doi: 10.1371/journal.pone.0161929 (PMC5063340; doi:10.1371/journal.pone.0161929)
Supplement: S2 Table — (DOCX) [file pone.0161929.s003.docx]

| Model number | Model | AICc | Weights |
| --- | --- | --- | --- |
| 1 | yi ~ 1 + Salinity group + Temperature | 409.90 | 6.07e-01 |
| 2 | yi ~ 1 + Salinity group + pH + Temperature | 411.36 | 2.92e-01 |
| 3 | yi ~ 1 + Salinity group + Water type + Temperature | 415.84 | 3.10e-02 |
| 4 | yi ~ 1 + Temperature | 416.08 | 2.76e-02 |
| 5 | yi ~ 1 + Salinity group + WT + pH + Temperature | 417.37 | 1.45e-02 |
| 6 | yi ~ 1 + pH + Temperature | 417.79 | 1.17e-02 |
| 7 | yi ~ 1 + Water type + Temperature | 417.88 | 1.12e-02 |
| 8 | yi ~ 1 + Water type + pH + Temperature | 419.39 | 5.26e-03 |
| 9 | yi ~ 1 + Salinity group | 432.53 | 7.36e-06 |
| 10 | yi ~ 1 + Salinity group + pH | 434.03 | 3.48e-06 |
| 11 | yi ~ 1 + Salinity group + Water type | 438.20 | 4.33e-07 |
| 12 | yi ~ 1 | 438.81 | 3.20e-07 |
| 13 | yi ~ 1 + pH | 439.93 | 1.82e-07 |
| 14 | yi ~ 1 + Salinity group + Water type+ pH | 440.36 | 1.47e-07 |
| 15 | yi ~ 1 + Water type | 440.48 | 1.39e-07 |
| 16 | yi ~ 1 + Water type + pH | 442.03 | 6.39e-08 |

**Model selection table for Zr using showing model structure, AICc and weights for each model, accounting for combinations of the four moderator variables included in the full model.**
